# From Awake to Minimalist Spontaneous Ventilation Thoracoscopic Mediastinum Surgery: How Far Are We?

**DOI:** 10.3390/jcm14238396

**Published:** 2025-11-26

**Authors:** Alexandro Patirelis, Vincenzo Ambrogi, Eugenio Pompeo

**Affiliations:** 1 Unit of Thoracic Surgery, Department of Surgical Sciences, University of Rome Tor Vergata, 00133 Rome, Italy; alexandro.patirelis@hotmail.it (A.P.); ambrogi@med.uniroma2.it (V.A.); 2 Division of Thoracic Surgery, Tor Vergata University Polyclinic, 00133 Rome, Italy

**Keywords:** awake thoracic surgery, spontaneous ventilation thoracic surgery, non-intubated thoracic surgery, mediastinum surgery, video-assisted thoracic surgery

## Abstract

Spontaneous ventilation (SV) video-assisted thoracic surgery (VATS) is aimed at offering less invasive alternatives to equivalent procedures under tracheal intubation with mechanical ventilation (MV) and its benefits have shown encouraging results in lung surgery. In addition, there is also growing interest in SV-VATS in mediastinum surgery. The rationale of SV in simpler mediastinum procedures is that MV anesthesia could be considered avoidable if SV anesthesia protocols could provide similar or even better results. On the other hand, for other indications involving more delicate patient subgroups, SV-VATS is aimed at offering a more rapid recovery with less anesthesia-related risks of cardio-respiratory complications. Based on encouraging initial results, SV is also being proposed for more demanding surgical procedures, including tracheal resection and esophagectomy. However, SV mediastinum surgery also implies contraindications, potential disadvantages and peculiar physiopathologic issues which must be clearly acknowledged. This perspective is aimed at providing a critical overview of the current knowledge about SV for mediastinum surgery, with a particular emphasis on the last 10 years of data about thymectomy, biopsy of mediastinal masses, thoracic sympathectomy, tracheal resection, pericardial window and esophagectomy.

## 1. Background

The history of thoracic surgery under spontaneous ventilation (SV) has its roots in the beginning of the previous century, when surgeons started to operate on awake subjects under local anesthesia. The driving force behind this practice originated from experience accrued during World War I, which showed the possibility that humans might survive despite a traumatic open pneumothorax [1]. Most of the thoracic surgery procedures at that time regarded the lung, pleural and chest wall. Nevertheless, SV thoracic surgery could also be applicable to the mediastinum. In 1954, Vischnevsky reported a pioneering experience of 400 surgical procedures, which included even esophageal surgery, performed under SV, by regional anesthesia in awake subjects [2].

At the beginning of the 1950s, the introduction of double-lumen tubes allowing one-lung mechanical ventilation (MV) [3] revolutionized thoracic anesthesia and rapidly became the gold standard. Advantages of intubation with single-lung MV include deep sedation, achievement of safe airway control and tailored ventilation of a single lung, allowing for surgical maneuvering within a nearly immobile anatomic field. Nonetheless, scientific data progressively underlined potential adverse effects, including risks of airway injury due to tracheal intubation and the so-called ventilator-related lung injury, which entailed barogenic, volugenic, and biogenic traumas [4]. These adverse effects are likely to become dangerous, particularly in patients with specific pathologic conditions, including huge masses in the anterior-superior mediastinum or those with autoimmune, neuromuscular disorders, such as myasthenia gravis.

As it has been shown for lung surgery [5], SV-VATS is re-emerging as a less invasive strategy in mediastinum surgery as well, claiming potential advantages for selected cohorts. Indications, which initially included the biopsy of mediastinal masses [6], thymectomy [7] and sympathectomy [8], have now progressively broadened to other surgical procedures, including mediastinum tumor excision, pericardial window and even tracheal and esophageal surgery. Overall, there was a gradual shift from procedures performed in fully awake patients to anesthesia protocols entailing target-controlled sedation, assuring unconsciousness with maintenance of SV, the adoption of supraglottic devices, such as laryngeal mask, and regional anesthesia by intercostal or paravertebral block or thoracic epidural analgesia. As a result, these strategies have been variably denominated as awake, nonintubated, tubeless or minimalist thoracic surgery. In particular, in terms of minimalist thoracic surgery, we are referring to VATS procedures entailing target-controlled sedation with the maintenance of SV, single surgical access, and adoption of advanced surgical technology, such as motorized staplers and energy devices, and minimized use or avoidance of any chest drainage.

The rationale for the application of SV in mediastinum surgery is that it may result in fewer complications [6], earlier mobilization, faster post-operative recovery and more rapid discharge than similar procedures carried out by intubation and MV [8]. However, relative contraindications, including patients with morbid obesity (body mass index > 30), coagulopathy unsuitable for thoracic epidural catheterization, or a surgical plan with expected significant technical difficulties due to anatomically complex conditions or large masses requiring radical excision, must be taken into account [5]. Moreover, peculiar physiopathologic issues of the mediastinum are related to the central anatomic position, the presence of important anatomic structures and its strict contiguity to both pleural cavities. Furthermore, it is recommended that SV mediastinum surgery is performed by well-trained teams in which anesthesiologists are able to rapidly convert to tracheal intubation even in lateral decubitus, whereas surgeons must operate facing intermittent lung and mediastinum movements induced by maintained rhythmic diaphragmatic contraction [5].

Despite several articles about different SV mediastinum surgeries having been published so far, no review has yet summarized the current state of the art about this topic. This perspective review has the following objectives:

- To analyze available data about different mediastinum surgeries performed under SV, with emphasis on published reports in the last 10 years.
- To describe some key anesthesiologic and surgical technical aspects.
- To describe the main reported outcomes, including comparisons with equivalent procedures under intubation with single-lung MV.
- To critically analyze the advantages and limitations of SV in mediastinum surgery.
- To highlight future perspectives and potential areas of investigation in this evolving field.

## 2. Thymectomy, Thymomectomy, and Other Mediastinum Tumor Excision

Anatomic thymectomy may be required in patients with autoimmune myasthenia, a neuromuscular disorder based on the development of antibodies directed against the acetylcholine receptor at the neuromuscular junction. In this context, medical therapy has been shown to be positively affected by thymectomy [9].

On the other hand, thymomas and other mediastinum tumors include a heterogeneous group of neoplasms optimally cured by surgical excision in the majority of the cases [10]. Notably, a thymoma is computed tomography (CT)-detectable in about 20% of myasthenic patients whereas about 30–50% of thymomatous patients are associated with autoimmune myasthenia [11,12].

Whatever the surgical indication for mediastinal tumor excision, advancements in surgical techniques have progressively shifted the mainstay approach from median sternotomy to minimally invasive strategies, such as video-assisted thoracic surgery (VATS) [13] and robotic-assisted thoracic surgery (RATS) [14]. This ensures a smaller incision, fewer post-operative complications, faster recovery and comparable long-term outcomes [15]. Conversely, minimally invasive approaches cannot achieve optimal exposure of the mediastinum, particularly when dealing with the superior part of the anterior compartment. As such, surgical visualization and maneuvering are hindered by the progressively reduced anatomic space in close proximity to the thoracic inlet [16].

The adoption of SV anesthesia is deemed a further step towards a global minimally invasive surgical management. The first report of transternal thymectomy under SV in awake patients is that of Tsunezuka et al. in 2004 [17]. Afterwards, Al-Abdullatief et al. reported a case series including 25 patients undergoing SV thymectomy for myasthenia gravis by VATS or median sternotomy [7].

Over the past decade, the interest in thymectomy or mediastinum tumor excision under SV anesthesia has progressively grown. Several studies explored its feasibility and outcomes (Table 1).

In all instances, target-controlled sedation was obtained with propofol infusion and monitored using bispectral index. Regional analgesia was assured by epidural catheterization [18,24,25], paravertebral block [23], or intercostal block [20,23], usually by a combination of lidocaine and ropivacaine. Additional oxygen was delivered by a laryngeal mask. Thymectomy, especially if associated with the removal of the perithymic fatty tissue in accordance with the criteria of an extended excision, might lead to the opening of both mediastinal pleurae. This phenomenon could impair lung ventilation due to the development of a bilateral pneumothorax. In these instances, a laryngeal mask may help provide supplemental oxygen, facilitate carbon dioxide removal and allow non-invasive positive pressure ventilation [26]. Multi-port unilateral VATS was the preferred access, with no particular preference about the side of access [20,22,24]. In 2017, our group reported on a small case series of patients undergoing thymomectomy by SV uniportal VATS [25]; afterwards, a similar larger series was published by Liu et al. [23]. Furthermore, in recent years, subxiphoid access under SV proved a wide exposure of the anterior mediastinum and avoidance of intercostal nerve damage with lesser post-operative pain [18,19,21].

According to the available data, thymectomy and mediastinum tumor excision by SV-VATS proved to be feasible and safe. Jiang et al. reported no deaths or major complications out of a total of 239 patients, with only a single case of conversion to general anesthesia and thoracotomy because of pleural adhesions [24]. Huang et al. performed this surgery in a cohort of patients with impaired lung function (forced expiratory volume during the first second <70%) with only five Clavien Dindo I complications [20], mimicking results already shown in similar cohorts with lung surgery [27,28]. The comparison between similar procedures carried out by MV anesthesia has shown no difference in operative time [20,21,23,24]. Conversely, Liang et al. reported that both operative and anesthesia times were significantly lower by SV-VATS [22]. Regarding short-term outcomes, as compared to MV, the SV group showed an earlier return to oral feeding [23], less pain [23,24], and a shorter post-operative hospitalization time [22,24].

When dealing with myasthenic patients, who are at risk of developing myasthenic crisis post-operatively in 6–34% of instances [19], the avoidance of muscle relaxants with SV anesthesia led to a zero-incidence rate [24]. Overall, the procedure proved effective in terms of both the completeness of thymectomy and clinical improvements [18,19,25].

### Controversial Issues

As compared with MV-VATS, the greater technical difficulty, due to lung and mediastinal movements or to coughing reflex during surgery under SV should be considered, particularly when dealing with vessel isolation and cutting. Moreover, temporary hypoxemia and/or hypercapnia may occur more frequently during SV-VATS, particularly in technically demanding surgical procedures with prolonged operative time. As a result, the available data, which is still based only on retrospective studies, should be considered insufficient to draw any conclusions about the superiority of SV-VATS in terms of post-operative outcomes.

## 3. Biopsy of Mediastinal Masses

Undetermined anterior mediastinal masses with radiologic features of benign cysts, mature teratomas or early-stage thymomas, commonly lead to direct surgical excision. As far as huge masses radiologically attributable to lymphomas [29], VATS is often employed due to its minimally invasive nature and the optimal visualization of mediastinal compartments, as well as the capability of retrieving adequate tissue specimens for precise histo-immunophenotyping. In addition, VATS allows for the drainage of associated pleural–pericardial effusions and multiple simultaneous biopsies of neighboring structures.

One limitation of VATS is the current need for general anesthesia under one-lung ventilation, which can be followed by life-threatening adverse events such as severe airway obstruction and even cardiovascular collapse, particularly in patients with bulky anterior mediastinal masses [30]. In fact, during standard anesthesia entailing the use of muscle relaxants, tracheal intubation and MV, large mediastinal masses may compress at weaning mediastinal structures leading to complications that increase operative mortality and prolong hospital stay [31].

The mechanisms of cardiovascular instability in this setting have been experimentally identified, while those of central airway obstruction remain poorly defined and largely uninvestigated [32].

In order to avoid surgical procedures in these high-risk patients, some authors proposed CT-guided needle biopsy as an alternative non-surgical diagnostic tool. Unfortunately, the diagnostic yield by this procedure proved sub-optimal and the risk of complications was low but not negligible. A recent meta-analysis revealed an overall diagnostic yield of 92%, although the presence of a high percentage of lymphomas in the evaluated studies increased non-diagnostic results [33]. In accordance with these findings, previous investigations reported a diagnostic yield ranging from 56% to 74% when considering only lymphomas [34,35]. Consequently, the European Society for Medical Oncology still recommends surgical biopsy for the diagnosis of suspected lymphoma for mediastinal masses, given its near-100% diagnostic yield [36,37].

**Figure 1:**
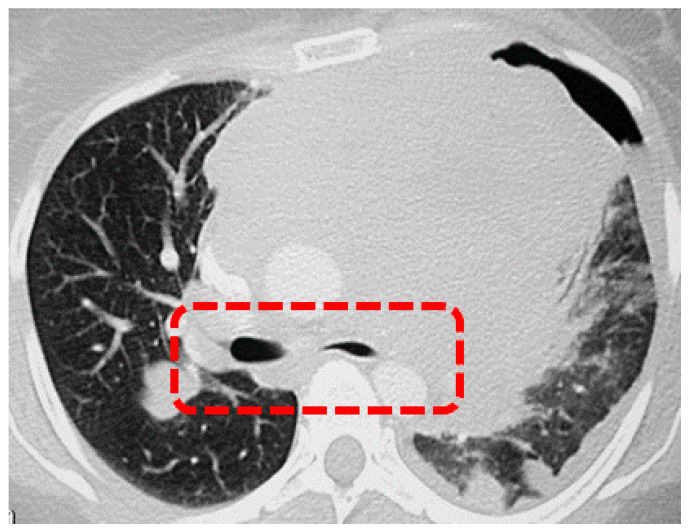
Reduction in major airways cross-sectional area due to anterior mediastinal mass ab extrinseco compression (highlighted in the red box).

The pathophysiology related to mediastinal masses is still not totally understood. The mass behaves as a space-occupying lesion in the mediastinum, competing with vital anatomic structures, such as the trachea and main stem bronchi, the great vessels and the heart. A reduction in tracheal cross-sectional area above 50% due to an ab estrinseco compression (Figure 1) is associated with a higher risk of peri-operative complications, including difficulty during post-operative weaning from MV [32,38]. In fact, anesthesia and particularly neuromuscular blockers may alter the precarious balance between the tumor and these structures [38]. The loss of SV after induction decreases the transpleural pressure gradient, which usually distends the intrathoracic airways avoiding their collapse. Furthermore, it has been assumed that MV in patients with central airway obstruction may increase post-stenotic turbulence of inhaled gases, reducing alveolar ventilation [32].

In contrast with previous findings, Hartigan et al. reported a series of cases wherein 17 patients with bulky mediastinal masses underwent awake intubation and monitoring changes in airway caliper with bronchoscopy [39]. They observed no significant changes from baseline in the mean airway patency after induction, in spite of the increase in patency after neuromuscular blockade and positive pressure ventilation. Nevertheless, this study does not refer to surgery and did not assess risks related to ventilator weaning, which usually represents the most critical time period for respiratory complications in these patients.

In this complex setting, the correct combination of high diagnostic accuracy and procedural safety seems to be facilitated by surgical biopsy under SV-VATS. Our group was the first to report in 2010 on SV-VATS biopsy for bulky anterior mediastinal masses in fully awake patients [6]. At the time, thoracic analgesia was based on an epidural catheter at the T4–T5 level or intercostal block with a mixture of lidocaine and ropivacaine. Surgery was usually performed through a single incision, and the side was chosen according to the peculiar anatomic distribution of the mass. Following access into the pleural cavity, multiple biopsies were taken by spoon-shaped forceps in the most representative targeted areas. It is worth noting that by this surgical method, our group reported a 100% diagnostic yield with no mortality or peri-operative cardiorespiratory adverse events.

As far as other studies published about this topic within the last 10 years are concerned, only one study published by Tacconi et al. in 2016 compared, by propensity score matching, the results of 24 patients who had undergone a minimalist uniportal SV-VATS mediastinum biopsy after epidural anesthesia or intercostal injection of lidocaine 2% plus ropivacaine 7.5%, to an equivalent group of patients who underwent biopsy by MV-VATS [40]. In this study, the overall diagnostic yield was 100%, whereas the lower incidence and severity of post-operative complications and higher PaO2/FiO2 values occurred in the SV-VATS group as compared to MV-VATS.

Han et al. have recently claimed the feasibility and safety of SV-VATS in a pediatric cohort of 92 patients, including 26 mediastinal mass biopsies with no mortality or respiratory complications and a mean hospital stay of 3.4 days [41].

### Controversial Issues

Despite the encouraging physiologic rationale and promising anecdotal data, mediastinum mass biopsy by SV-VATS still remains an under-investigated topic. Furthermore, due to the compression of the central airways, an emergency conversion to tracheal intubation could be challenging for anesthesiologists without adequate training in SV-VATS.

## 4. Thoracic Sympathectomy

Primary palmar hyperhidrosis is an idiopathic disorder characterized by excessive sweating of the hands, armpits, and/or feet. These symptoms could cause serious social, emotional and professional problems, and affect quality of life. Most patients are usually adolescents or young adults [42]. Several non-surgical treatments, such as botulinum toxin, iontophoresis or systemic anticholinergic agents have been proposed as palliative non-surgical treatments [43]. Nevertheless, VATS thoracic sympathectomy, with the interruption of the upper thoracic sympathetic chain, usually at the T2–T4 level by clipping or excision, is currently the only effective and potentially definitive treatment, which has resulted in high patient satisfaction [44].

The first study comparing one-stage bilateral thoracic sympathectomy in SV-VATS versus MV-VATS came from our group in 2005 [8]. In this study, patients in the SV-VATS group remained fully awake during the procedure with supplementary oxygen administered through a face mask and regional anesthesia obtained with ropivacaine-based intercostal block. The excision of the T3–T4 sympathetic thoracic chain was first accomplished on the right side and entailed the placement of a chest tube at the end of the operation. Afterwards, the same procedure was accomplished on the left side; at end-procedure, the right drainage was removed, and full left lung re-expansion was monitored by video assistance without any drainage placement. Comparisons with MV-VATS results showed shorter operative and hospitalization times and higher satisfaction, as assessed by quality of life questionnaires.

The adoption of SV for sympathectomy is justified by the peculiar population target of the procedure, which is young and healthy. Sympathectomy is intended to treat the negative psychological impact of excessive sweating, which may severely affect the social life of these subjects. The avoidance of intubation with the maintenance of SV does not expose patients to potential MV-related side effects, may be considered simpler and makes post-operative recovery and discharge faster [8].

A literature review conducted over the past decade has identified four relevant studies [45,46,47,48]. Three of these [45,46,47] compared SV-VATS versus MV-VATS thoracic sympathectomy. All the procedures were performed in a one-stage bilateral fashion. Analgesia was based on an intercostal block, and patients underwent surgery under target-controlled sedation with propofol and remifentanil/sufentanil. Chen et al. reported the use of a laryngeal or face mask to deliver supplemental oxygen [45], whereas Shao et al. preferred the adoption of a high-flow nasal cannula [48]. Concerning the surgical approach, VATS with a small incision along the anterior or the midaxillary line was preferred by all the authors, with the exception of Chen et al., who adopted an innovative transareolar 5 mm incision in their cohort of male patients [45]. At end-procedure, a chest tube was initially placed in all patients and then immediately removed after the intrathoracic application of negative pressure [49] to promote lung re-expansion. The outcome analysis showed that SV-VATS thoracic sympathectomy is safe since no mortality nor major post-operative complications were recorded. Only one patient required conversion to general anesthesia and MV due to pleural adhesions [46]. When taking into account all the reported series, out of a total of 298 patients, only 5 of them (1.7%) required a chest tube insertion. In 3 of these cases, the drainage was placed during surgery [48] whereas in the other 2 chest tube insertion was necessary during the post-operative period due to pneumothorax shown on chest X-ray [45,48]. The comparison with equivalent procedures performed under MV showed no difference in global operative time. Conversely, patients in the SV-VATS group started feeding sooner [46], complained of less pain [45,46] and had shorter hospitalization times [45,46,47]. In particular, Caviezel et al. emphasized that 90% of the procedures under SV might be performed in a one-day surgery setting, reflecting a significant reduction in global costs [47]. The analysis of long-term outcomes has shown no difference in quality-of-life assessment and satisfaction [45,47], as well as in symptom resolution and incidence of compensatory hyperhidrosis [45].

### Controversial Issues

Thoracic sympathectomy by SV-VATS proved to be a safe procedure. Nevertheless, MV-VATS also proved to be associated with low morbidity [45] due to the typical healthy young population. As a result, large, controlled trials are warranted to suggest non-inferiority and to explore the potential benefits of SV-VATS thoracic sympathectomy as compared to MV-VATS.

## 5. Tracheal Resection

Airway resection and reconstruction may be required for a tracheal tumor or post-intubation benign stenosis. Regardless of the etiology, pathologic conditions involving the trachea may become highly symptomatic with unpaired ventilation and poor quality of life. Useful diagnostic tools include bronchoscopy, magnetic resonance imaging, CT and positron emission tomography (PET) scans. As a result, the resection and reconstruction of the affected tracheal tract may be indicated for radical oncologic treatment, symptom relief and quality of life improvement [50].

Standard surgical management involves the adoption of general anesthesia with endotracheal intubation to shelter the airway and allow for mechanical ventilation. Thus, cross-field intubation, entailing the insertion of a sterile endotracheal tube into the distal trachea, is commonly performed to assure lung ventilation during tracheal resection and reconstruction [51]. However, in certain patients, such as those with significant comorbidities or difficult airway anatomy, general anesthesia through endotracheal intubation may not be feasible or desirable. Moreover, cross-field intubation may hinder a precise anastomosis due to the encumbrance created by the tube itself [52]. Alternatively, high-frequency jet ventilation can be employed [53], although sometimes it may induce barotrauma and hypoxygenation [54]. Extracorporeal membrane oxygenation has also been anecdotally adopted in complex instances, but its application is hindered by the elevated invasiveness [55].

Therefore, within the range of available approaches, SV tracheal surgery has emerged in recent years as a viable, less invasive alternative. The rationale is that air can ventilate distal airways by flowing through the surgical incision into the tracheal opening [56]. The lack of a tracheal tube makes the resection of the lesion more rapid and tracheal suturing during the reconstruction phase could become easier, thus decreasing operative time and risk of complications. Lastly, SV could give the surgeon the opportunity to test the nerves’ integrity during the procedure [56].

The first series of about 20 upper tracheal resections and anastomosis for benign stenosis in SV awake patients by median sternotomy, was reported by Macchiarini et al. in 2010 [57]. He recorded no need for conversion to MV or post-operative complications. Despite these encouraging results, SV tracheal surgery is still anecdotal and mainly based on simple case reports [58,59,60]. Among these, particularly noteworthy are the first ones entailing uniportal VATS [61] and uniportal RATS [62].

To the best of our knowledge, only three studies included a wider population. Zhou et al. reported their first 51 cases of SV upper tracheal surgery through a cervical incision with or without partial sternotomy [63]. Patients were sedated, and local analgesia was performed through a cervical plexus block and thoracic epidural catheterization in case of sternotomy. In all patients, a laryngeal mask was placed pre-operatively. There was no operative mortality. During surgery, two patients required temporary jet ventilation due to oxygen desaturation without interrupting SV. Three patients were converted to cross-field intubation due to pneumothorax for the incidental opening of the pleural cavity or because of uncontrolled cough. In this series of cases, the morbidity rate was 13.7% and was mainly constituted by pneumothoraxes in 11.8% of the cases.

Liu et al. reported their series of 33 patients undergoing intrathoracic tracheal resection by SV-VATS [64]. The patients received a laryngeal mask (27/33) or an oro-tracheal tube (6/33) before the beginning of surgery for additional oxygen supplementation, even if SV was always maintained during the resection phase. They reported a 27.3% conversion to cross-field intubation whereas morbidity included one case of atrial fibrillation and one case of anastomosis dehiscence.

To date, Jiang et al. reported the only study comparing tracheal or carinal surgery by SV-VATS versus MV-VATS [65]. These authors employed target-controlled sedation with propofol as well as a laryngeal mask for oxygen delivery. They reported in the SV-VATS group no need for conversions to intubation, shorter operative times regarding both tracheal end-to-end anastomosis and carinal reconstruction, faster post-operative recovery and a lower morbidity.

### Controversial Issues

Taking into account the currently available data, tracheal resection by SV anesthesia proved to be feasible and fast. Nonetheless, the experience is still limited and a non-negligible risk of conversion has been reported, suggesting the need for further thorough investigation. In addition, the adoption of SV may be associated with a potential risk of hypoxemia and/or hypercapnia, and in this setting, the lack of airway control may be perceived as harmful, both by surgeons and anesthesiologists.

## 6. Pericardial Window

Pericardial effusion is defined as an abnormal accumulation of fluid into the pericardial cavity, ranging from the absence of symptoms to cardiac tamponade [66]. Pericardial window entails the removal of a portion of the pericardium to allow for the effusion to drain into the pleural cavity. This procedure is employed for recurrent pericardial effusion and has a double purpose, to reduce both the mass effect and hemodynamic imbalance and obtain a diagnostic biopsy [67].

The surgical procedure is commonly performed by VATS, which is usually better tolerated than open surgery [68]. Nevertheless, the more complex pharmacological management required by general anesthesia with MV may represent a risk factor in these frail patients. Indeed, patients with pericardial effusion can rapidly decompensate following induction because of a combination of factors, such as vasodilatation and diminished preload, leading to a quick deterioration of cardiovascular function, possibly culminating in an acute cardiac tamponade [69]. For these reasons, pericardial surgery represents a promising and yet underexplored indication amongst SV surgery mediastinum procedures.

Despite the theoretical advantages of SV-VATS, only a few case reports or short case series have been published [68,70]. We found a unique series of 20 patients undergoing pericardial window by SV uniportal VATS [71]. The patients were mildly sedated with propofol during the procedure and a nasal cannula or face mask was adopted for supplemental oxygen. In this study there were 2 operative mortalities, although these were attributed to other associated medical comorbidities and not directly to the surgical procedure. Overall, mortality and morbidity proved similar to previously published data, suggesting that this surgical procedure is feasible and relatively safe. Moreover, the authors reported a shorter hospitalization (a median of 1 day) compared to previous data from the literature, allowing for rapid post-operative chemotherapy treatment in the case of malignant effusion.

### Controversial Issues

Pericardial window under SV may be relatively simple and rapid to perform. Thus, it might provide significant theoretical benefits related primarily to the avoidance of intubation, curarization, and MV in patients who are often critically ill and unstable. Nevertheless, the available data are currently based on anecdotal reports, so it is still premature to draw any conclusions, particularly in comparison with MV-VATS.

## 7. Esophagectomy

Esophagectomy for radical treatment of esophageal cancer under SV is still a poorly investigated topic. The historical background includes the pioneering work of Vischnevsky in 1954. He reported about 193 esophageal surgery procedures, and 111 of them were performed in fully awake patients by multiple regional anesthesia, including wide vagal and phrenic nerve blocks [2].

The current gold standard in the radical treatment of esophageal cancer is the McKeown operation, consisting of triple access, thoracic, abdominal and cervical, to accomplish a subtotal esophagectomy and esophageal replacement by a gastric tube with cervical esophagogastric anastomosis. In this setting, VATS has been increasingly employed to accomplish the isolation of the thoracic esophagus and lymphadenectomy. However, despite the adoption of VATS, esophagectomy by tracheal intubation and MV still results in a relatively high rate of respiratory complications, such as pneumonia (17.1%), prolonged MV (18.3%) or the need for reintubation (16.2%) [72,73]. In this context, SV could avoid the risks associated with intubation, thus enhancing post-operative recovery and reducing morbidity [74].

A review of the literature identified only two studies addressing this topic. Namely, a small case series of three patients [75] and a case–control study consisting of a total of 33 patients, 20 of them belonging to the SV group [76]. In both studies, target sedation, a laryngeal mask and vagal block were employed. Furthermore, SV was maintained during the thoracic section only, whereas synchronized intermittent mechanical ventilation was preferred during abdominal and cervical sections. The study comparing SV versus MV esophagectomy revealed that discontinuous SV was not inferior in oxygenation values, and the higher values of carbon dioxide did not cause ventilatory impairments. Moreover, surgical times were comparable, no cases of conversion to MV nor thoracotomy were necessary and no patient died in either group [76].

The results of SV during esophagectomy are encouraging, although preliminary. Notably, a Chinese group recently started a clinical trial comparing SV versus MV in VATS esophagectomy for esophageal cancer with an estimated enrollment of 500 patients (Clinical Trial NCT07104838).

### Controversial Issues

In addition to the limited evidence on SV-esophagectomy, the performance of this technically demanding surgical procedure may be further complicated by lung and mediastinal movements. Moreover, the risks of bilateral pneumothorax, as well as of hypercapnia due to long operative times, should be taken into account. Thus, SV-VATS esophagectomy should be considered highly investigatory and only an option for surgical teams with a consolidated expertise in SV strategies.

## 8. Limitations

The main limitation was the impossibility of performing a systematic review with meta-analysis due to the limited number of published reports, which often included small retrospective cohorts with no comparison with MV control groups. In addition, reported experiences addressed heterogeneous topics and entailed the adoption of different anesthesia protocols, which represent ineludible sources of bias and strongly limit the robustness of the analysis. Finally, the lack of randomized trials entailing SV mediastinum surgery further contributes to the limited generalizability of our findings.

## 9. Future Perspectives

There is growing interest in SV mediastinum surgery. Published reports during the last 10 years, even though still anecdotal in some specific fields, suggest a non-inferiority or even superiority of these strategies when compared to equivalent procedures carried out by MV-VATS. In particular, the adoption of SV-VATS for thymic surgery, mediastinum tumor excision, mediastinum mass biopsy and thoracic sympathectomy appears to be associated with lower morbidity rates and a faster post-operative recovery, which may contribute to facilitating early recovery after surgery (ERAS) protocols [77].

Within this clinical scenario, for simpler indications such as thoracic sympathectomy, MV anesthesia may reveal overmanagement, provided that simpler SV anesthesia protocols may offer similar or even better results. On the other hand, for other indications involving more delicate patients with bulky mediastinum biopsy, recurrent pericardial effusion, thymectomy or mediastinum tumor excision, SV-VATS may offer a safer alternative with fewer anesthesia-related risks of cardio-respiratory complications, although scientific evidence in this regard is still insufficient. In addition, even for more technically demanding surgical procedures, such as tracheal surgery, SV-VATS and RATS are being proposed as a safe alternative to MV. Finally, regarding esophageal surgery, the data currently available is too scant to draw any reliable conclusions, although the feasibility and safety of VATS esophagectomy seem to potentially benefit as well from SV-anesthesia.

The current literature reveals a marked heterogeneity in anesthetic management, including differences in airway devices, sedation depth, regional analgesia techniques and potential adverse effects (Table 2). This variability reflects the evolving nature of SV anesthesia for mediastinum surgery and underlines the need for standardized protocols. The development of consensus guidelines and dedicated training programs for both surgical and anesthetic teams will help ensure safety and reproducibility and will eventually facilitate the implementation of SV-VATS in mediastinum surgery.

Another interesting field of investigation could be the potential beneficial effects of SV on immunologic function after surgery, as already hypothesized with lung surgery [78]. When dealing with oncologic mediastinum surgery, a lower immunologic impairment in the early post-operative period might play a protective role against recurrence and improve survival. Furthermore, it could have a function in the mitigation or avoidance of symptoms and the development of complications after surgery in patients with compromised health status, multiple comorbidities and severe non-neoplastic disease such as autoimmune myasthenia gravis.

Additional novel technology, including the use of artificial intelligence, will help identify optimal candidates for each anesthesia strategy and will probably contribute to achieve more accurate peri-operative management of patients requiring SV-VATS.

## 10. Conclusions

This perspective is the first to report a comprehensive review of different mediastinum surgeries performed under SV. Preliminary findings suggest that SV could be a safe alternative to MV, offering potential advantages, such as simplified anesthesia management, reduced post-operative morbidity and faster recovery for both simple and more complex procedures, as anecdotally reported. These findings may contribute to promote less invasive anesthesia strategies within ERAS protocols. Additional evidence from large randomized controlled studies is warranted to help standardize anesthesia protocols, clarify which mediastinum surgeries can be safely performed using SV strategies and which may offer comparable or superior outcomes to procedures carried out by intubation with MV anesthesia.

## Figures and Tables

**Table 1:** Studies about SV-VATS for thymectomy, thymomectomy and other mediastinum tumor resections published between 2015 and 2025.

First Author	Year	Country	Study Design	Number of Patients	Surgical Access	Comparison with MV-VATS (SV vs. MV)	MG Patients
Hartert [18]	2022	Germany	Case series	3	Subxiphoid	No	Yes
Liu [19]	2021	China	Case series	10	Subxiphoid	No	Yes
Huang [20]	2020	China	Retrospective	32	Triportal	Yes (15 vs. 17)	No
Mao [21]	2020	China	Retrospective	40	Subxiphoid	Yes (21 vs. 19)	Yes
Liang [22]	2019	China	Retrospective with PSM	198	Biportal	Yes (55 vs. 55)	Yes
Liu [23]	2019	China	Retrospective	225	Uniportal	Yes (96 vs. 129)	No
Jiang [24]	2018	China	Retrospective	104	Triportal	Yes (36 vs. 68)	Yes
Pompeo [25]	2017	Italy	Case series	3	Uniportal	No	Yes

MG: myasthenia gravis; MV: mechanical ventilation; PSM: propensity score matching; SV: spontaneous ventilation; VATS: video-assisted thoracic surgery.

**Table 2:** Adverse effects of SV mediastinum surgery according to the type of procedure.

	Mediastinum Tumor Excision	Mediastinum Mass Biopsy	Thoracic Sympathectomy	Tracheal Resection	Pericardial Window	Esophagectomy
Risk of hypoxemia	+	+/−	+/−	++	+/−	++
Risk of hypercapnia	+	+/−	+/−	++	+/−	++
Lack of safe airway control	−	+	−	+/−	−	+
More difficult surgical maneuvering due to mediastinum movement	+	+/−	+/−	+/−	−	+
Risks of bilateral pneumothorax	++	−	−	+	−	+

Legenda: −, no risk; +/−, low risk; +, real risk; ++, high risk.
